# Maintaining quality of life after major lung resection for carcinoid tumor

**DOI:** 10.1186/s13019-023-02435-7

**Published:** 2023-11-14

**Authors:** Riad Abdel Jalil, Farah A. Abdallah, Zeinab Obeid, Mohamad K. Abou Chaar, Ahmad Khaled Harb, Tariq Bassam Shannies, Ahed El-Edwan, Hussam Haddad, Azza Ghraibeh, Ahmad Abu-Shanab

**Affiliations:** 1 Department of Thoracic Oncology, King Hussein Cancer Center, Queen Rania Al Abdullah Street, P.O. Box 1269, Amman, 11941 Jordan; 2 Department of Research, King Hussein Cancer Center, Amman, Jordan; 3 Department of Surgery, King Hussein Cancer Center, Amman, Jordan; 4 Department of Anesthesia, King Hussein Cancer Center, Amman, Jordan; 5 Department of Pathology and Laboratory Medicine, King Hussein Cancer Center, Amman, Jordan; 6 Department of Radiology, King Hussein Cancer Center, Amman, Jordan

**Keywords:** Bronchopulmonary carcinoid, Pulmonary function, Quality of life, Post-operative pain, Peri-operative pulmonary care program.

## Abstract

**Background:**

Pulmonary carcinoid is a rare diagnosis with surgery remaining the standard treatment of choice. However, resection may impact patients’ daily activities due to decreased lung volume reserve and postoperative pain. Our study aims to compare the impact of different types of surgical resection on the post-operative quality of life with the application of a strict peri-operative pulmonary care program.

**Methods:**

Patients who underwent surgery for bronchopulmonary carcinoid tumors in a tertiary cancer center between August, 2017 and March, 2020 were identified and demographic data was collected. Patients were contacted via phone for the qualitative and quantitative assessment of pain and quality of life, utilizing the Arabic version of Short-form McGill Pain Questionnaire and Activity of Daily Living (ADL) instrument respectively. Lung reserve was assessed before and after surgery. Statistical analysis used Chi-Square for categorical variables and ANOVA for continuous variables.

**Results:**

A total of 16 patients underwent different type of resection. The majority were male (n = 10; 63%) with a mean age of 44 years (19–81). Most common clinical stage was stage I (n = 12, 75%) with typical carcinoid features recorded in more than half of the cases (n = 11, 69%). Almost all patients underwent surgical excision (n = 15, 94%) with negative resection margin and no major post-operative complications. Bilobectomy was the most frequent procedure (n = 6, 40%) and video-assisted thoracoscopic surgery (VATS) was utilized in 8 patients (50%). Expected changes were recorded in pre- and postoperative pulmonary function test with an average drop of 10 in FEV1 and 14 mL/min/mmHg in DLCO. The majority of patients (n = 15, 94%) were totally independent doing daily activities. Mild intermittent pain was found in 7 patients (44%) who scored an average intensity of 1.6 out of 10.

**Conclusions:**

Excellent long-term outcomes can be achieved following surgical resection of pulmonary carcinoid tumors with little to no effect on patients’ lung function and quality of life in regard to performance status and post-operative pain when a good peri-operative pulmonary, physical rehabilitation, and pain management programs are adopted and strictly implemented.

## Background

Pulmonary carcinoids account for 27% of carcinoid tumors and less than 1% of all lung cancers [1, 2]. Around 80% of lung carcinoids are found centrally in the main bronchi, while 20% may be located within the periphery extending to the parenchyma. The anatomic location of these usually determines the management approach. They characteristically present with pulmonary symptoms including cough, recurrent lung infection, hemoptysis, shortness of breath and wheeze. Surgery remains the gold standard treatment which may include radical lung resection as well as minimally invasive access and parenchymal sparing bronchial resection [3]. However, lung preserving surgery is the treatment of choice when applicable for typical and anatomical resection is recommended for atypical carcinoid tumors [4]. Additionally, endobronchial debulking procedures are reserved for tumors located in the central airways and for patients who can’t tolerate surgical resection. Patients with surgical resected typical carcinoid tumors have a 5-year survival rate approaching 92%, with a 5% chance of tumor recurrence. With regards to surgically resected atypical carcinoids, the figures for 5 survival rate and recurrence drop to 70% and 25%, respectively [3].

The anatomical location of these carcinoid tumors within the bronchus and bronchioles poses a threat to the surrounding parenchyma. The majority of bronchial carcinoids are malignant and capable of metastasizing. This is more distinctive of atypical carcinoids which display aggressive behavior and spread both locally and distally. Therefore, surgical resection might lead to shortness of breath and exercise induced fatigue knowing that the decrease in lung function is clinically irrelevant. Additionally, resection may impact patients’ ability to undertake daily activities due to decreased lung reserve and postoperative pain. Morality with pulmonary carcinoid following resection is around 4.1% with a high overall 15-year survival of 77.5% [5]. Therefore, assessment of postoperative quality of life and the presence of chronic postoperative pain (CPSP) is crucial. Chronic postoperative pain (CPSP) has been found in up to one third of patients following lung resection [6, 7]. These patients were found to have considerably worse physical function and a diminished quality of life [7].

The aim of this study is to analyze postoperative quality of life, pain and pulmonary function of patients at our institution when a good peri-operative pulmonary and physical rehabilitation programs as well as pain management service were adopted and strictly implemented on these surgical patients. To our knowledge, no study to date specified the quality-of-life evaluation for lung carcinoid patients following surgical resection with extensive pain score evaluation.

## Methods

From a prospectively maintained electronic health record registry, data encompassing demographics, tumor characteristics and treatment outcomes was gathered for patients who underwent surgical treatment for localized pulmonary carcinoid tumors between (August 2017- March 2020) at the King Hussein Cancer Center (KHCC). The study was approved by the institutional review board (IRB) with the number 19KHCC146. Data of tumor characteristics included histological subtype, mitotic rate (per high power field), Ki67, staging, lymphovascular invasion, largest tumor diameter (cm) and the presence of positive lymph nodes. All patients underwent primary curative surgical resection without neoadjuvant chemoradiotherapy. None of the patients were diagnosed with carcinoid syndrome. Oral consent to conduct the quality of life and pain questionnaires was obtained through an IRB approved phone script.

### Peri-operative Pulmonary Care Bundle Implementation

We designed the perioperative pulmonary care bundle on multiple stages starting at the time of diagnosis. If a patient was an active smoker, cessation guidance and counseling was provided. On admission, the patient was educated and trained by a respiratory therapist about aspiration prevention, deep breathing exercises, incentive spirometer use, and chest physiotherapy. Minimally invasive approach was utilized when feasible. Early post-operative mobilization was accompanied by adequate pain control achieved by specialized pain team [8].

### Pulmonary Function Test

A pulmonary function test (PFT) was conducted pre-operatively and repeated after surgery to document a change in lung capacity and ventilation using values of Forced Expiratory Volume (FEV1) and diffusing capacity of the lungs for carbon monoxide (DLCO). Post-operative PFT was performed at an average of 2 years.

### Quality of Life Assessment

After obtaining an oral consent, the Arabic version Activity of Daily Living (ADL) instrument [9] was conducted by one of the research personnel through the phone. This tool was used to measure the adequacy of performance of 6 daily functions each scaled as dependent, independent or intermediate. The first half of the tool which includes bathing, dressing, going to the toilet yielded a Cronbach’s α of 0.90 and the second half transferring (movement), continence, and feeding was found to have Cronbach’s α of 0.65. The scores of the ADL section of the questionnaire were calculated using the ADL Scoring Manual. The total score of each subgroup is summated to calculate 0 points (very dependent) 1–5 points (partially dependent) and 6 points (completely independent). Patients were contacted after an average of 2 years post-surgery.

### Pain Assessment

The Arabic version of Short-form McGill Pain Questionnaire (SF-MPQ) by Terkawi et al. [10] was performed via a phone call after obtaining an oral consent. The tool consists of 2 subscales; sensory perception and affective dimension of pain (Cronbach’s α = 0.85). Sensory pain assessment includes throbbing, shooting, stabbing, sharp, cramping, gnawing, hot burning, aching, heavy, tender, splitting. Similarly, affective pain consists of tiring/exhausting, sickening and fearful. The pain is scored from 0 to 3; 0 no pain, 1 mild, 2 moderate and 3 severe. Same as before, pain assessment was performed at an average of 2 years postoperatively.

### Statistical analysis

All statistical analysis has been conducted on SPSS version 24. The findings of the study have been presented as descriptive statistics. The relationship between categorical data has been tested using the Chi-square test, while continuous data associations were evaluated using ANOVA. A value of ≤ 0.05 (CI = 95%) is considered statistically significant.

## Results

Our study included 16 patients with the majority being male (10, 63%) and a mean age of 44 years (± 19.53). Patients stratified according to stage as: stage I (12, 75%) and stage II (4, 25%). Histopathological features showed typical carcinoid in 11 patients (69%) and atypical histology in 5 patients (31%). Only one patient (6%) received adjuvant chemotherapy. Two patients (13%) were found to have positive lymph nodes, each one of them with only one hilar lymph node involved. No major postoperative morbidity or mortality was observed. Pain management is maintained by shoulder block, intercostal block, and paravertebral block intra-operatively, followed by close observation by the pain team who administer patient controlled analgesia then transition into oral pain medication. All patients were alive when contacted with no local or distant tumor recurrence (Table 1).

**Table 1:** Demographic and clinical data

Variable		Number of Patients n = 16
Age (years)		43.50 (± 19.5)
Gender	Male	10 (63%)
	Female	6 (38%)
Comorbidities		
	Diabetes Mellitus	2 (13%)
	Hypertension	7 (44%)
	Other	5 (31%)
Histologic Subtypes		
	Typical	11 (69%)
	Atypical	5 (31%)
Staging		
	Stage I	12 (75%)
	Stage II	4 (25%)
Adjuvant Chemotherapy		1 (6%)
Surgical Approach		
	VATS^Ω^	11 (48%)
	Open	12 (52%)
Negative Resection Margin (R0)		15 (94%)
Lymph Node Involvement		2 (13%)
Complications		5 (31%)

Ω Video-assisted thoracoscopic surgery

**Fig. 1:**
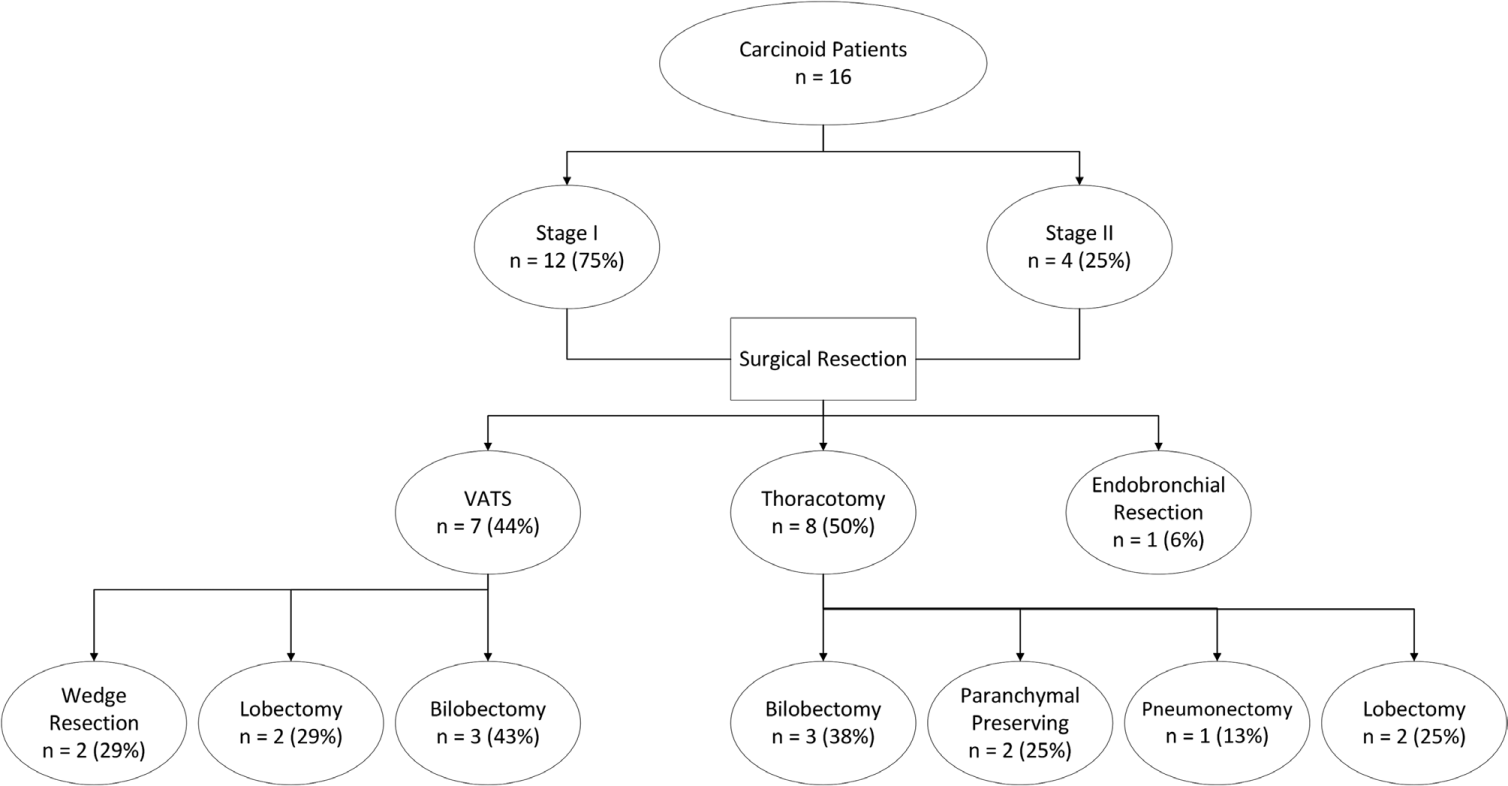
CONSORT Diagram describing studied group

Fifteen of the patients (94%) underwent complete tumor resection which included: parenchymal preserving, wedge resection, lobectomy, bilobectomy, sleeve lobectomies and pneumonectomy. Bi-lobectomies were performed on tumors located at the bifurcation of middle and lower lobe bronchi with an average size of 2.3 cm. A single patient required endobronchial debulking is an 82-year-old with multiple comorbidities who cannot tolerate lung resection. Complete microscopic resection with negative margin was achieved in all cases. The use of minimally invasive video-assisted thoracic surgery (VATS) was reserved for 8 patients (50%) who were deemed eligible (Fig. 1).

The collective mean preoperative DLCO was 105.07 as compared to 95.92 postoperatively (p = 0.437). In terms of FEV1, the mean preoperative value was 88.00 ± 20.785 and became 75.75 ± 19.82 postoperatively (p = 0.133). Changes in FEV1 and DLCO were not statistically significant (Table 2).

**Table 2:** Pulmonary function test pre-operatively and post operatively

	Preoperative FEV_1_%	Postoperative FEV_1_%	Change (%)	Preoperative DLCO%	Postoperative DLCO%	Change (%)
Minimum	48	38	↓ 21%	63	73	↑ 16%
Maximum	134	111	↓ 17%	192	146	↓ 24%

As described by SF-MPQ, mild intermittent pain was found in 7 patients (43.7%) scored an average intensity of 1.625 out of 10. The mean sensory pain was 0.085 out of 3 and affective pain was 0.078. The most common type of pain described by patients was cramping in 4 patients scored as mild to moderate. This was followed by moderate to severe throbbing mean in 3 patients (Table 3).

**Table 3:** Results from the short-form McGill Pain Questionnaire (**SF**-**MPQ**)

Pain	Minimum	Maximum	Mean	Median	Std. Deviation
**Throbbing**	0	3	0.44	0	0.964
**Shooting**	0	0	0.00	0	0.000
**Stabbing**	0	1	0.06	0	0.250
**Sharp**	0	2	0.13	0	0.500
**Cramping**	0	2	0.31	0	0.602
**Gnawing**	0	0	0.00	0	0.000
**Hot burning**	0	0	0.00	0	0.000
**Aching**	0	0	0.00	0	0.000
**Heavy**	0	3	0.31	0	0.793
**Tender**	0	0	0.00	0	0.000
**Splitting**	0	2	0.19	0	0.544
**Tiring/exhausting**	0	3	0.19	0	0.750
**Sickening**	0	3	0.19	0	0.750
**Fearful**	0	3	0.19	0	0.750

Using the ADL tool, we found that 15 out of 16 patients (94%) were totally independent and only one patient (6%), who had undergone endobronchial debulking due to his multiple comorbidities, was partially dependent.

## Discussion

Based on our data, carcinoid tumor resection modality does not seem to have an effect on pulmonary function test if patients were conditioned properly in the peri-operative period. All of our patients, with exception to one were found to have that surgery had no effect on their daily activities and function. Those who reported minimal pain after surgery, did not require any form of analgesia and were able to function normally.

An interest in patient-centered outcomes is witnessing an escalation in healthcare management. It is pivoted on long-term outcomes like health-related quality of life, residual dysfunctions, and chronic pain rather than focusing on endpoints such as morbidity and mortality. Hence, we call for informing patients about the effect of lung resection on postoperative residual function and quality of life (QoL).

A number of studies investigate risk factors leading to a decline in postoperative QoL after lung resection, predicting and evaluating models to predict postoperative QoL [11, 12]. Advanced age, extent of lung resection, and neoadjuvant chemotherapy showed a significant decline in the physical aspects of QoL. However, no significance was reported in terms of gender, tumor staging, or postoperative complications [13]. We did not find any significant change in quality of life in our patient cohort.

With adequate peri-operative pain control, chronic post-operative pain can be avoided. Chronic post-surgical pain (CPSP) is defined as persistent pain three months after thoracic surgery, different in nature from preoperative pain, and is not caused by other conditions [14]. The majority of pain is found to be neuropathic in nature, which the literature has attributed CPSP to possible damage of the intercostal nerve which is seldom avoided even with the emergence of VATS [15]. Only 3–16% of affected patients will have moderate to severe symptoms [16]. Pleuritic chest pain in nature is described as burning at the incision dermatomes, exacerbated by light touch, temperature changes, and shoulder movements [17]. Risk factors that have been attributed to CPSP are age, female gender, severe acute post therapeutic pain, high analgesic consumption, history of chemotherapy or radiotherapy administration, and the extent of the surgery [18, 19].

Variability exists in the radicality of surgical approaches for resection of bronchopulmonary carcinoids. Current options including parenchymal preserving; where the bronchus is resected and anastomosed while the lung remain intact, wedge resection, lobectomy including sleeve lobectomy, bilobectomy and pneumonectomy. When tumors are located at the bifurcation of lobe bronchi, a sleeve resection to preserve any of the lobes is necessary. The effect of pulmonary resection on lung function is attributed to the magnitude, site and modality utilized for resection. Moreover, postoperative pulmonary rehabilitation and the prevention of lung fibrosis influence the extent of diminished lung function. Keenan et al. found that less radical resection of pulmonary tumors offers preservation of lung function compared to a more aggressive approach [20]. Similarly, Lee et al. noted a significant relationship between the number of resected segments and the change in FEV1 [21]. However, regardless of the extent of resection, the Japanese Association of Chest Surgery and American College of Chest Physicians preoperative risk assessment of lung function must be carried out to ensure values of FEV1 and DLCO are > 60% [22, 23].

## Conclusion

Following surgery to remove pulmonary carcinoid tumors, long-term outcomes including higher survival rates, controlled symptomatology, and decreased likelihood of tumor recurrence may be achieved. Our data reported a minimal effect of extensive lung resection on postoperative lung function, quality of life and pain when we adopt a good peri-operative pulmonary and physical rehabilitation programs as well as pain management service. Despite the limited number of patients analyzed, we believe our findings support the use of aggressive surgical resection for this rare entity.

## Data Availability

The datasets used and/or analyzed during the current study are available from the corresponding author on reasonable request.

## Abbreviations

VATS: Video assisted thoracic surgery
CPSP: Chronic postoperative pain
KHCC: King Hussein Cancer Center
PFT: Pulmonary function test
FEV1: Forced Expiratory Volume
ADL: The Activity of Daily Living
SF-MPQ: Short-form McGill Pain Questionnaire
QoL: Quality of life
ERAS: Enhanced Recovery After Surgery
